# Modeling the Comovement of Entropy between Financial Markets

**DOI:** 10.3390/e20060417

**Published:** 2018-05-30

**Authors:** Petre Caraiani

**Affiliations:** Institute for Economic Forecasting, Romanian Academy, 050711 Bucharest, Romania; caraiani@ipe.ro; Tel.: +40-724-415-392

**Keywords:** entropy, financial markets, comovement

## Abstract

In this paper, I propose a methodology to study the comovement between the entropy of different financial markets. The entropy is derived using singular value decomposition of the components of stock market indices in financial markets from selected developed economies, i.e., France, Germany, the United Kingdom, and the United States. I study how a shock in the entropy in the United States affects the entropy in the other financial markets. I also model the entropy using a dynamic factor model and derive a common factor behind the entropy movements in these four markets.

## 1. Introduction

Financial networks have become a widely used tool for practitioners and academics in finance. Their main appeal comes from their methodological versatility. Using them as a tool can lead to a more profound understanding of financial phenomena. Researchers have used networks in a variety of contexts related to financial markets, including modeling the interrelationships between stocks, investors, or banks; analyzing contagion; detecting the structure of financial markets; and designing macroprudential policies and early warning methods for financial systems. It is almost impossible to summarize the literature here. However, a few notable papers are those of References [1,2,3,4,5,6,7,8,9].

In the context of applications of networks to financial markets and institutions, a very useful approach has proven to be the use of entropy-based measures. Several studies have focused on the use of entropy as a variable that can reveal the state of the market or a tool that can be used to explore the relationship between different stocks.

An innovative study has been carried out in Reference [10], which studied the causal relationship between the largest 187 companies in the world using transfer entropy. Using this approach, they were able to identify the influences between the companies in this sample, and they found a central role for insurance companies in the United States and Europe.

An exciting line of research has proposed the use of entropy as a means to characterize the state of a financial market. Reference [11] found it is possible to construct an entropy-based index using singular value decomposition applied to the matrix of correlations of financial stocks in the United States. This entropy measure has predictive power for the future dynamics of stock markets. Further studies using a similar approach to Reference [11] confirmed this finding; see the study in Reference [12] for analysis of the Shenzhen stock market based on the entropy derived using singular value decomposition of stock market correlation matrices.

More recent applications of entropy in the context of financial networks comprise different approaches to the use of entropy. An example is Reference [13], which has applied a measure of Shannon entropy at a multiscale level on the stock market. This concept of entropy has predictive power for stock market dynamics both in the short and in the long run. In an approach based on transfer-entropy, Reference [14] studied the causal relationship between stocks and commodities in time, over the last 20 years. Their results indicate that commodities are decoupled from equity markets, although the energy equity sector and energy futures comove.

Another recent use of entropy in the context of financial markets is due to Reference [15], which applied the permutation entropy approach to the case of Chinese stock markets to investigate whether the complexity (or the degree of information) increases or decreases during stock market crashes. They found that permutation entropy declined during the last financial crisis in the case of the key market indices for Chinese stock markets.

In this paper, I extend the previous studies by considering a novel perspective regarding the relationship between stock markets. I build on previously developed approaches from literature to measure the entropy of financial networks (the matrices of correlations of financial stocks) for four developed stock markets (namely, the United States, the United Kingdom, France, and Germany). I further analyze the relationship between the time-varying entropy measures for these countries by using modern multivariate time series techniques.

This paper is not the first to consider comovement between international stock markets of financial networks, although the literature is rather scarce. For example, a recent study [16] analyzed the time-varying relationship between stock market indices in 83 countries. While their analysis is very pertinent, the present study differs through the fact that it rather focuses on deriving entropy measures for each of the four developed stock markets included here, and it further analyzes the comovement between the entropy measures, rather than studying single indices for each country. A similar valuable study has been carried in Reference [17] for 56 countries using daily data for stock market indices and currencies. Other studies focused on financial market spillovers within a network context are those in References [8,9].

The present paper applies modern multivariate time series techniques, including a dynamic factor model, and it allows one to uncover the dynamics interrelationships between the entropy measures constructed for the countries in the sample. Thus, this paper proposes a new approach in understanding the spillovers and comovement between the entropy measures in developed financial markets.

The paper proceeds as follows. The next section introduces the methods used to build the entropy-based measures of financial networks. It also presents the time series techniques used to study the comovement between the entropy measures developed for each of the countries in the sample. The third section introduces the data. The fourth section derives the entropy measures for each of the included financial markets and studies their comovement. Section five discusses the results and draws conclusions.

## 2. Methodology

Here, I introduce the methodology and the main quantitative instruments used throughout the paper.

### 2.1. Correlation Networks of Stocks

The analysis in this paper uses financial networks derived from correlation matrices between the components of the main stock market indices in each of the selected countries. There are various ways to construct financial networks based on correlations or other means to detect relationships (including Granger causality or transfer entropy). Reference [18] reviews the different methods to compute correlations among stocks for financial networks.

This paper uses the following formula to determine the (simple or Pearson) correlations among two stocks:

(1) $$\rho_{i,j}=\frac{cov(r_{i},r_{j})}{\sigma_{r_{i}}\sigma_{r_{j}}},$$

where I denote by $r_{i}$ the return of the a given stock *i*. Furthermore, I use $\sigma_{r_{i}}$ for the standard deviation of the return of stock *i*. I compute the return of a stock *i* using the logarithmic difference:

(2) $$r_{i}\left(t\right)=\log P_{i}\left(t\right)-\log P_{i}\left(t-1\right).$$

In this case, $r_{i}\left(t\right)$ is the return of the stock *i* at moment *t*, and $P_{i}\left(t\right)$ denotes the value of the stock *i* at time *t*.

I derive correlation matrices of stocks for each of the selected stock market indices in the sampled countries. This matrix of stocks can be used as the underlying adjacency matrix to derive the financial network. However, the focus of the paper is on the determining the corresponding entropy for the already determined matrices of correlations. I first present the singular value decomposition approach, and then detail how I determine the entropy on the basis of the latter.

### 2.2. Singular Value Decomposition

Singular value decomposition (denoted by SVD hereafter) can be applied to any matrix. In this context, I apply SVD to decompose the correlation matrices of stocks. While SVD can be applied to a given correlation matrix at a given point in time, the methodology here will be based on a rolling window approach, in order to derive time-varying measures of entropy.

I consider the general case of a matrix *A*, with dimensions m×n. This matrix can be decomposed using SVD in the following manner:

(3) $$A=USV^{T}.$$

Here, the matrix *U* has dimensions m×k and the matrix *V* has dimensions dimensions k×n. The matrices *U* and *V* are chosen such that the matrix *S* has the form:

(4) $$S=\mathrm{diag}(\lambda_{1},\lambda_{2},\ldots ,\lambda_{k}),$$

where *k* is determined by k=min(m,n). The matrix *S* has two essential properties, namely its elements are positive and they are ordered in a decreasing order.

### 2.3. Singular Value Decomposition-Based Entropy

I now introduce a global measure of networks used throughout the paper, i.e., the singular value decomposition-based entropy. Based on the SVD method presented above, one can construct an entropy measure. The entropy measure employed here is based on the contribution of Reference [19], which is an extension of the reference study [20].

To derive the entropy, I start from the singular values which are extracted in the previous step using SVD (as detailed above) and denote these values by $\lambda_{k}$. One can normalize these values via:

(5) $$\lambda_{k}=\frac{\lambda_{k}}{\sum \lambda_{k}}.$$

In the following step, the formula below is applied:

(6) $$SvdEn=-\sum \lambda_{k}\ln\left(\lambda_{k}\right).$$

Here, I denote the singular value decomposition based entropy by SvdEn (I maintain this notation throughout the paper). Newer developments with respect to computing the entropy of networks can be found in Reference [21].

## 3. Data

While data for the most financial market indices in the world is accessible, it has proven to be more difficult to identify stock market indices for which full historical data on their components were available. It is true that daily data is available for all main stock market indices in the paper. However, while daily data could be indeed found for most components of these indices, it is critical for the paper that there are no missing observations. Any missing observations would lead to the necessity of eliminating that day from all components of the main stock market indices. This should be done not only for one particular country, but in the end for all, since I model the four measures of entropy from the countries in the sample.

Some remarks should be further made. First, the paper focuses on long enough time series in order to allow for a relevant historical analysis using the dynamic factor approach (that is, the time span should be as long as possible). Second, previous research, see Reference [11], has shown that using daily or monthly data for computing the singular value decomposition leads to similar qualitative and quantitative results.

For these reasons, I focused on a smaller set of financial countries for which data at monthly frequencies were available. I selected data for the components of the main stock market indices in the United States (US, Dow Jones Industrial Index), the United Kingdom (UK, FTSE), France (CAC40), and Germany (DAX30). The monthly data sample is between January 2000 and December 2017. It must be underscored that the methodology can be easily extended to daily data.

## 4. Results

### 4.1. Computing the Singular Value Decomposition-Based Entropy

I introduce here the derivation of the entropy using singular value decomposition. Since I am interested in this paper in deriving a time-varying value of entropy, I implement a moving window technique. The size of the window is 40 observations, corresponding to about three and a half years. The choice of the window size is based on two criteria: the window should long enough to uncover the dynamics of the entropy (computing correlations requires a sufficient number of observations), while its size should not be too large (which would smooth the changes in entropy). Singular value decomposition is computed for each instance of the sliding window on the available matrix of correlations. I slide the window in each period by one observation to the right. In the end, I obtain a time series for SVD-based entropy for each of the four financial markets in the study.

Appendix A shows the derived entropy measures along the corresponding stock market indices, shown in their first difference. A simple visual inspection shows that there is a lot of variation in entropy around the crisis period, starting with 2008. Particularly, there is a significant drop in the entropy for each case right before the crisis, while during the financial crisis, the entropy spikes significantly. This pattern of remarkable changes in entropy around financial turbulence has been also noticed in the studies of References [11,15]. My results essentially confirm these findings.

A second significant change is around 2015, when in each case the entropy drops. This drop corresponds to a stabilization in the financial markets, as the special interventions program of central banks had ended and economic growth had recovered. Again, the behavior is uniform among the markets included here.

### 4.2. Does the SVD-Entropy Have Predictive Power?

The descriptive analysis from the previous section suggests a connection between entropy and the state and dynamics of financial markets. To further test this quantitatively, I consider the Granger causality test between entropy and the main stock market index in each country.

Table 1, Table 2, Table 3 and Table 4 show the results of the Granger causality test. I considered the entropy taken as a difference (since the index series are not stationary). In general, I found that there is evidence of causality from the entropy to the log-difference of stock market returns. These findings confirm previous studies for the United States and China, which were already mentioned in the introduction. They underline the predictive power of the entropy measures with respect to the aggregate stock market indices in each country.

### 4.3. Analyzing the Comovement between Entropy Measures

In this section, I focus on the key contribution of this paper: proposing a method to analyze the comovement between the constructed indices for entropy using singular value decomposition. I use two approaches: a vector autoregressive (VAR) model, and a dynamic factor model (DFM), using for both the first difference of the entropy indices in the four stock markets. Each of the models has a different function. I use the VAR model to quantify the spillovers from one entropy index to the entropy indices of the other countries. The role of the dynamic factor model is to uncover whether there is an underlying factor that can explain the movements of the individual entropy measures.

#### 4.3.1. VAR Model

In this section, I use a VAR model to study the spillovers between the entropy measures in the four selected countries. One would expect that an (unexpected) increase in the entropy in the United States stock market, for example, would affect the entropy measures from the other countries in the sample.

I estimate a model with the following specification:

(7) $$y_{t}=c+A_{1}y_{t_{1}}+\ldots +A_{p}y_{t-p}+u_{t}.$$

Here, $y_{t}$ is the vector of the series of interest (in this particular case, the vector of entropy measures) with dimension k×1, *c* is a constant vector of dimension k×1, the matrices $A_{i}$ are matrices of coefficients of dimension k×k while $u_{t}$ is the vector of innovations, of dimensions k×1. It is further assumed that $u_{t}$ is white noise, i.e., $E(u_{t})=0$; $E(u_{t}u_{t}^{\prime })=\Sigma$, where Σ is the covariance matrix; and $E(u_{t}u_{s}^{\prime })=0$, for t≠s.

**Figure 1:**
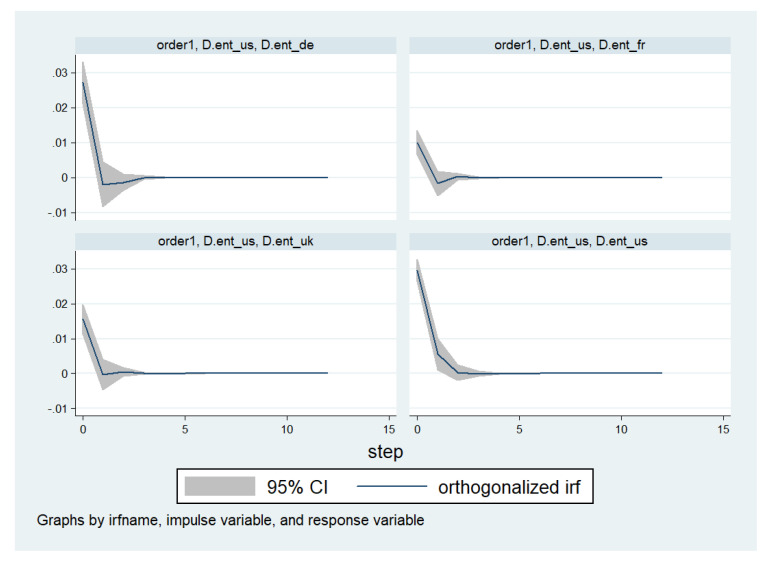
Vector autoregressive (VAR) model: Effects of a shock in entropy on the United States stock market; D.ent_us is the first difference of entropy in United States, D.ent_fr the first difference of entropy in France, D.ent_de the first difference of entropy in Germany and D.ent_uk the first difference of entropy in UK.

I select only one lag, following the information criteria that allow discrimination between the different lag lengths, see Appendix B. The model is estimated using the entropy measures in growth specification (since in this specification they are stationary). Below, I present the main result of interest, namely the impact of a shock in the entropy of United States stock market on the entropy measures of the other financial markets, see Figure 1. The computation of the impulse response function uses an ordering of variables following basic intuition, with the entropy measure in the US first, followed by that in the UK, and then by the entropy measures in France and Germany.

Figure 1 shows a few interesting features. First, the impact is rather short-lived, both when affecting the entropy in the United States or when affecting the entropy in the other financial markets. Second, although the impact of innovation in entropy in the United States on the entropy in other markets is initially positive, it also has a slight negative impact 1–2 lags afterward. These findings suggest that the spillovers from the entropy in the United States to the entropy in the other financial markets are not linear and might involve mechanisms which are not well studied.

#### 4.3.2. Dynamic Factor Model

In this section, I extend the comovement analysis and estimate a DFM model. The model specification is shown below:

(8) $$y_{t}=Pf_{t}+u_{t}$$

(9) $$f_{t}=A_{1}f_{t-1}+\ldots +A_{t-p}+v_{t}$$

(10) $$u_{t}=C_{t}u_{t-1}+\ldots +C_{t-q}u_{t-q}+\epsilon_{t}.$$

I denote again the vector of endogenous variables (the vector of entropy measures) by $y_{t}$. The first equation describes the dynamics of endogenous variables $y_{t}$ as being driven by $n_{f}$ underlying factors $f_{t}$. *P* is a matrix of parameters, while $u_{t}$ is a vector of disturbances. The second equation describes an autoregressive process for the underlying factors $f_{t}$. $v_{t}$ is a vector of disturbances, while the $A_{i}$ are matrices of parameters. Finally, the last equation describes the dynamics of the disturbances from the first equations, where the $C_{i}$ are matrices of parameters and $\epsilon_{t}$ a vector of disturbances.

**Figure 2:**
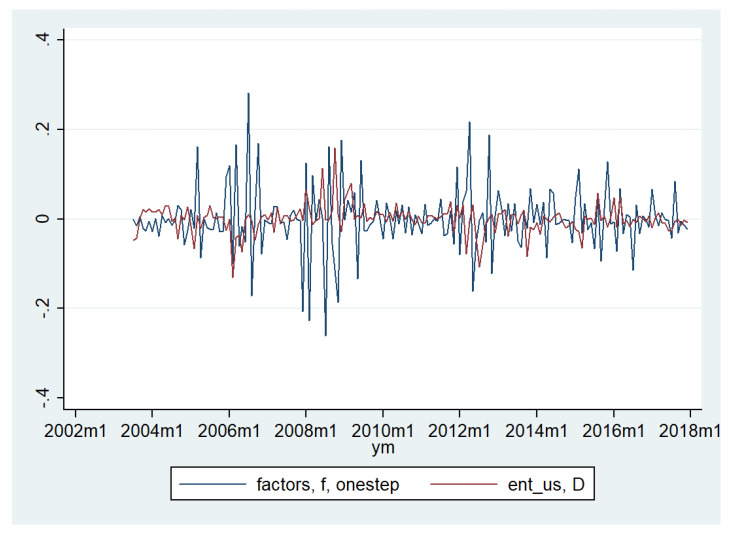
Dynamic factor model (DFM): Entropy for the United States stock market vs. DJIA index (Dow Jones Industrial Average). ent_us is the entropy in United States, taken as the first difference.

The role of the model is to uncover whether there is a common factor underlying the dynamics of the variables of the model, and if so how strong the factor is. I estimate a DFM with the entropy variables taken in their first difference. I use two lags in the specification. Figure 2 shows the dynamics of the underlying factor model versus the dynamics of the rate of change in the entropy measure for the United States. 

The behavior of the factor before and during the financial crisis shows that this factor has responded to the conditions in the financial markets. It has a lower value just before the crisis, while, once the financial crisis starts, there is higher growth in the value of the factor. The factor becomes also very volatily during this period. Since, by construction, the factor reflects common changes in the entropy in the selected financial markets, the results suggest a rather strong common factor that gives a measure of the international dimension of entropy.

The above figure also identifies another episode characterized by a larger fluctuation in the entropy, around 2012. This can be associated with the European sovereign debt crisis since it corresponds to the moment when the long term interest rates in Greece, Ireland, Portugal, and Spain had reached their maximum. The stock markets in France and Germany were most affected (from the sample of the four countries in this paper) due to the exposure of their banks to these countries. However, it is also possible that there were some consistent spillovers to the United Kingdom or the United States.

## 5. Discussion

The main purpose of this study was to extend the previous literature regarding the use of entropy in modeling financial networks. I considered several types of extensions. First, I have extended previous work regarding the study of financial networks through the use of entropy measures by considering a dataset of several developed financial markets. Deriving for the countries in this sample the entropy of the correlation matrices for the main stock market indices, I was able to show that the computed entropy measures have the potential the uncover the state of the markets. In each case, the entropy measure decreased just before the financial crisis from 2007–2008, while it increased dramatically once the crisis began. This finding is robust across the countries considered here.

A more significant contribution of this paper was, however, the use of time series techniques to study the comovement between entropy measures across countries. I have included two approaches. In the first approach, I have applied a vector autoregressive model, VAR, to study the spillovers between the entropies in the different financial markets. I found that the effects of an unexpected (shock) increase in the entropy of United States results in an increasing entropy in the other financial markets initially, but has slight negative effects over the next few periods. The effects are, however, short-lived.

In another application, I considered a dynamic factor model to uncover the common underlying factor for the four measures of entropy previously derived. Using the estimated dynamic factor model, I could uncover a common factor. It was found that the common factor varied with the conditions in the global markets, increasing as the financial crisis started. The common factor also seems to explain a significant share of the variation in the entropy measured for the financial markets in the United States. Furthermore, the common factor presented an increased volatility at the peak of the European sovereign debt crisis.

This resulting common factor could be thought of as a global entropy measure, or, at least, as a entropy measure for the developed markets. While the work here is exploratory, future studies could enrich this by adding emerging markets to the sample and deriving both a global entropy measure for developed markets and one for emerging economies.

The results in this paper contribute to a deeper understanding of the international dimension of informational comovement and spillover in financial markets. While there is a huge literature in finance dealing with international comovement for various financial dimensions, not much work has been done in the context of financial networks. Some attempts have been made to quantify the comovement of financial networks, however, the topic remains largely unexplored. Although a lot of work remains to be done, this study contributes to this promising line of research.

## Figures and Tables

**Table 1:** Granger Causality Test: France.

Lag Entropy Measure	Difference
1 lag	4.4120 **
2 lags	3.0309 *
3 lags	2.0704
4 lags	2.6570 **
5 lags	2.1805 *
6 lags	1.9988 *
12 lags	1.4698

Note: * denotes statistical significance of the *F*-test at 0.10 level; ** statistical significance at 0.05 level.

**Table 2:** Granger Causality Test: Germany.

Lag Entropy Measure	Difference
1 lag	2.8538 *
2 lags	1.8786
3 lags	1.3255
4 lags	2.6909 **
5 lags	2.1248
6 lags	2.2077 **
12 lags	1.5059

Note: * denotes statistical significance of the *F*-test at 0.10 level; ** statistical significance at 0.05 level.

**Table 3:** Granger Causality Test: United Kingdom.

Lag Entropy Measure	Difference
1 lag	2.7656 *
2 lags	1.4809
3 lags	1.0108
4 lags	2.4784 **
5 lags	2.016 *
6 lags	1.7967
12 lags	1.7188 *

Note: * denotes statistical significance of the *F*-test at 0.10 level; ** statistical significance at 0.05 level.

**Table 4:** Granger Causality Test: United States.

Lag Entropy Measure	Difference
1 lag	0.6132
2 lags	1.64
3 lags	1.6518
4 lags	3.1409 **
5 lags	2.4338 **
6 lags	2.0707 *
12 lags	1.6947 *

Note: * denotes statistical significance of the *F*-test at 0.10 level; ** statistical significance at 0.05 level.

**Figure A1:**
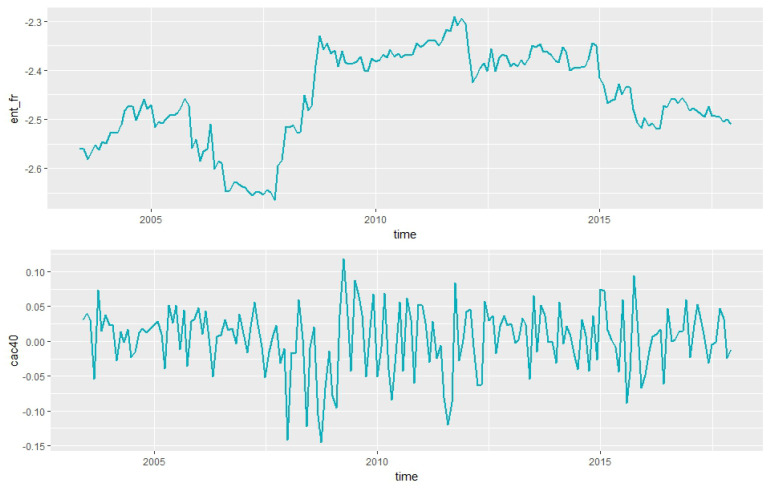
Entropy for France (ent_fr) and the CAC40 index.

**Figure A2:**
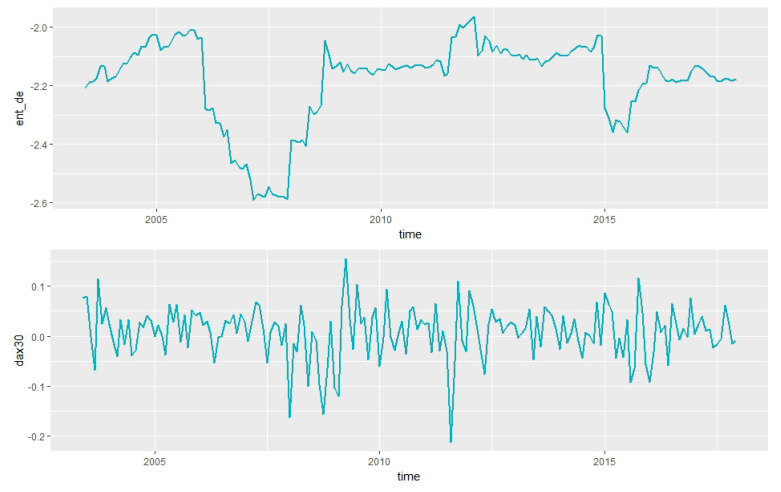
Entropy for Germany (ent_de) and the DAX30 index.

**Figure A3:**
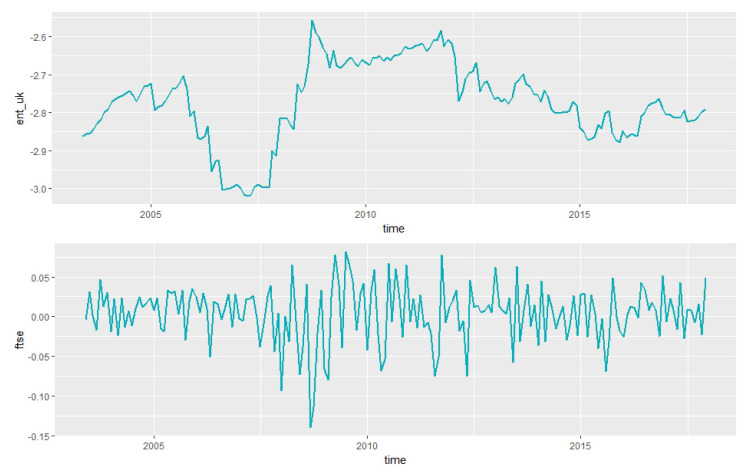
Entropy for the United Kingdom (ent_uk) and the FTSE index.

**Figure A4:**
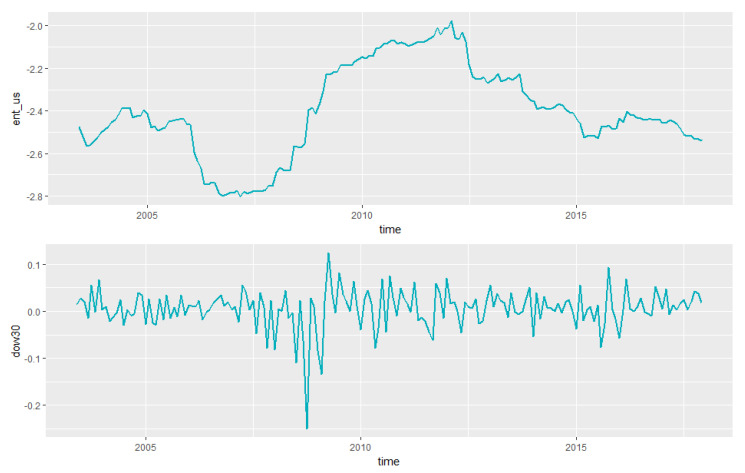
Entropy for the United States (ent_us) and the DJIA index.

## Abbreviations

The following abbreviations are used in this manuscript:

SvdEn	Singular value decomposition entropy
VAR	Vector autoregressive
DFM	Dynamic factor model

## Appendix A. Entropy Indices

**Table A1:** Unit Root Tests.

Country	Entropy (Index)	Entropy (Growth)	Returns Stock Market
France	−1.29	−5.87 ***	−4.72 ***
Germany	−2.50	−4.20 ***	−5.36 ***
United Kingdom	−1.86	−5.17 ***	−4.80 ***
United States	−1.80	−3.82 **	−4.99 ***

Note: ** denotes statistical significance of the unit root test at the 0.05 level; and *** at 0.01 level.

## Appendix B. Lag Order Selection in the VAR Model

**Table A2:** Selection-order criteria.

Lag	LL	LR	df	p	FPE	AIC	HQIC	SBIC
0	1601.43				$8.1\times 10^{-14}$	−18.7933	−18.7633 *	−18.7195 *
1	1621.79	40.729	16	0.001	$7.7\times 10^{-14}$ *	−18.8446 *	−18.6949	−18.4757
2	1632.04	20.491	16	0.199	$8.2\times 10^{-14}$	−18.7769	−18.5075	−18.1129
3	1647.87	31.668 *	16	0.011	$8.3\times 10^{-14}$	−18.775	−18.3857	−17.8158
4	1660.77	25.799	16	0.057	$8.6\times 10^{-14}$	−18.7385	−18.2295	−17.4842

Note: * denotes the selection of optimal lag length according to a certain informational criterion. Results From Stata.
